# Historical trends in survival of hospitalized heart failure patients: 2000 versus 1995

**DOI:** 10.1186/1471-2261-7-2

**Published:** 2007-01-16

**Authors:** Eyal Shahar, Seungmin Lee

**Affiliations:** 1 Division of Epidemiology and Biostatistics, Mel and Enid Zuckerman College of Public Health, The University of Arizona, Tucson, AZ, USA; 2 Department of Food and Nutrition, College of Human Ecology, Sungshin Women's University, Seoul, Korea

## Abstract

**Background:**

Population-based secular trends in survival of patients with congestive heart failure (CHF) are central to public health research on the burden of the syndrome.

**Methods:**

Patients 35–79 years old with a CHF discharge code in 1995 or 2000 were identified in 22 Minneapolis-St. Paul hospitals. A sample of the records was abstracted (50% of 1995 records; 38% of 2000 records). A total of 2,257 patients in 1995 and 1,825 patients in 2000 were determined to have had a CHF-related hospitalization. Each patient was followed for one year to ascertain vital status.

**Results:**

The risk profile of the 2000 patient cohort was somewhat worse than that of the 1995 cohort in both sex groups, but the distributions of age and left ventricular ejection fraction were similar. Within one year of admission in 2000, 28% of male patients and 27% of female patients have died, compared to 36% and 27% of their counterparts in 1995, respectively. In various Cox regression models the average year effect (2000 vs. 1995) was around 0.75 for men and 0.95 to 1.00 for women. The use of angiotensin converting-enzyme inhibitors and beta-blockers was associated with substantially lower hazard of death during the subsequent year.

**Conclusion:**

Survival of men who were hospitalized for CHF has improved during the second half of the 1990s. The trend in women was very weak, compatible with little to no change. Documented benefits of angiotensin converting-enzyme inhibitors and beta-blockers were evident in these observational data in both men and women.

## Background

Congestive heart failure (CHF), a common syndrome in the US [1], has been called the new epidemic of cardiovascular disease due to a recent increase in its prevalence [2,3] and the associated high mortality risk [4]. Trends in the public health burden of CHF are often attributed to three main factors: a shift in the age distribution of Western populations [5], favorable trends in survival after a myocardial infarction [6], and longevity of patients with hypertension [7-9].

Improved understanding of the pathophysiology of the failing heart coupled with new therapies should have led to improved survival of CHF patients in the last decade. In particular, beneficial drugs such as beta-blockers and angiotensin converting-enzyme (ACE) inhibitors assumed a dominant role in the management of the syndrome [10]. Nonetheless, it is still difficult to estimate the magnitude of survival trends in population-based samples.

We report here trends in mortality of hospitalized heart failure in a large metropolitan area in the Midwest region of the U.S.

## Methods

### Study design

This study was an epidemiological investigation of hospitalizations involving CHF among residents of metropolitan Minneapolis-St. Paul, Minnesota (a total of seven counties) [11]. The source population comprised over one million men and women (mostly Caucasians) who were served by 23 hospitals. Heart failure-related hospitalizations were identified from lists of discharge diagnoses, (*International Classification of Diseases, 9^th ^revision *428 and eleven other codes), from which patient-based sampling frames were constructed. Of the 23 hospitals, 22 agreed to participate. The single non-participating hospital was small, estimated to account for 1% of the patients. After stratifying on hospital and sex, samples of 50% and 38% of the records were reviewed in 1995 and 2000, respectively. (Changes in state and federal regulations concerning the use of medical records for research accounted for the smaller fraction in 2000.)

Trained study nurses abstracted information from the medical records and entered the data onto computerized forms. The nurses followed a manual of operation, resolved ambiguous information in consultation with the study physician (ES), and used a short screening form to exclude patients for whom a heart failure discharge code indicated a historical diagnosis, unrelated to the index hospitalization. To ascertain death following discharge from the hospital, the patient identifiers were linked to a statewide death certificate registry [6,12].

### Analysis

In the absence of a gold standard diagnosis of heart failure, numerous research groups have proposed various definitions for epidemiological studies and clinical trials. To allow for that diversity, six classification algorithms were applied to each record in the data set, adapting criteria used by six studies: the Framingham criteria [13], the Boston criteria [14], the Rotterdam criteria [15], the heart failure endpoint in the Antihypertensive and Lipid-Lowering Treatment to Prevent Heart Attack Trial (ALLHAT) [16], the National Health and Nutrition Examination Survey (NHANES) criteria [5], and the criteria proposed by the European Society of Cardiology [17]. For this analysis, a heart-failure related hospitalization should have met at least four of these six definitions (the majority).

Characteristics of patients in the two period cohorts (1995 and 2000) were compared by computing proportions, means and standard deviations, or medians and inter-quartile ranges (for skewed distributions.) Hazard ratios of death for the year effect and for various other variables were estimated by Cox regression. Covariates were selected on the basis of prior knowledge of their relation to mortality and their influence on the coefficient of the time variable. Finally, the associations of death with sex, age, and left-ventricular ejection fraction were estimated in year-specific Cox regression models. Point estimates and 95% confidence intervals are reported.

### Ethical considerations

The study was approved by the IRB of the University of Minnesota (IRB file 9711S00170) and the IRBs of participating hospitals in Minneapolis-St. Paul.

## Results

A total of 2,257 patients in 1995 and 1,825 patients in 2000 were determined to have had a CHF-related hospitalization. The male to female ratio was about 1.2:1 in both years. In both sexes, the two period cohorts displayed similar distributions of age, left-ventricular ejection fraction (when available) and length of hospital stay, but the 2000 cohorts had a somewhat worse cardiovascular risk profile – higher prevalence of ever-smoking, hypertension, diabetes, coronary disease, and previously diagnosed CHF (Table 1.) With few exceptions, patients hospitalized in 2000 were more likely than patients hospitalized in 1995 to be treated with ACE inhibitors, beta-blockers, and spironolactone during the hospital stay, and to be prescribed these drugs at discharge.

**Figure 1:**
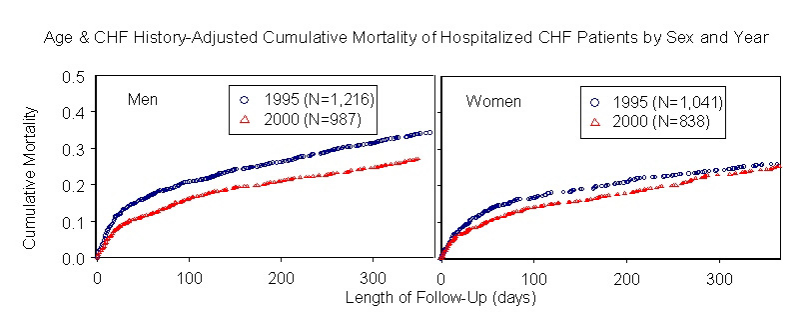
Age and CHF-history adjusted cumulative mortality of hospitalized CHF patients by sex and year.

In the first six months following a CHF-related hospitalization in 2000, 21% of male patients and 18% of female patients have died, as compared with 27% and 21%, respectively, in 1995 (Table 1). By one year of admission in 2000, 28% of male patients and 27% of female patients have died, compared to 36% and 27% of their counterparts in 1995, respectively. After taking age and history of CHF into account (Figure 1), the favorable secular trend remained apparent in men. Early separation of the cumulative mortality curves was evident in women as well, but there was no consistent divergence toward the end of the one-year follow up.

Table 2 shows the effect of the year of hospitalization on death, as estimated by several Cox regression models. Again, the secular trend was clear in men, though modest, and not so clear in women. As expected, having CHF prior to the index admission conferred greater hazard of death, whereas the prescription of ACE inhibitors and beta-blockers was associated with substantially lower hazard of death in the following year. Because only few patients received spironolactone in 1995, the hazard ratios (and 95% confidence intervals) were estimated only for 2000 patients: men, 1.02 (0.77–1.35); women, 0.70 (0.48–1.02). Prescription of digoxin at discharge was associated with lower hazard of death (HR = 0.72 in men and HR = 0.83 in women), but adding this variable to the models did not change the year effect (data not shown). Including diabetes status in these models did not materially affect the results, either.

Viewed differently, a weaker secular trend in women than in men should mathematically correspond to a weaker effect of sex on mortality in 2000 than in 1995. Indeed, as shown in Table 3, the hazard ratio of death comparing men to women has diminished from 1.41 in 1995 to 1.09 in 2000. The effect of age on death increased in both sexes whereas that of left-ventricular ejection fraction increased in men and decreased in women. In both years, missing ejection fraction was associated with poor survival, likely serving as a surrogate for disease severity.

## Discussion

It is often a challenge to estimate the magnitude of secular trends using epidemiological measures of cardiovascular disease. In particular, changes in the case mix and in diagnostic classification make it difficult to separate true changes in population-based samples from artificial components. Nonetheless, public health research naturally follows breakthrough in therapy to determine whether medical care has changed, and whether such a change has translated into detectable secular trends [18]. In this analysis of two surveillance years – 1995 and 2000 – we found modest survival improvement of male patients who were hospitalized for CHF, and little to no improvement in female patients. At least part of the favorable trend in survival of CHF patients may be attributed to greater use of well-tested drugs whose benefits were generally evident in this analysis as well (Table 2.)

Several studies, employing various designs and various sampling methods, have investigated secular trends in CHF survival in the US. The Framingham study reported improved survival of cohort members with CHF in successive decades since the 1950s [19]. The Rochester Epidemiology Project in Minnesota, reported no change in CHF survival in 1991 versus 1981; [20] overall improved survival between 1979 and 2000; yet a smaller gain for women than men [21]. Local and national studies of Medicare beneficiaries reported some or no improvement in mortality following hospitalized CHF during the 1990s [22,23], whereas one hospital-based study in the US reported modest, but significant, survival improvement particularly after 1998 [24]. Studies from Italy [25], Sweden [26], Scotland [27] and the United Kingdom [28] have generally found favorable trends in survival of CHF patients, paralleled by documented increased use of beta-blockers and ACE inhibitors during the 1990s [10].

Several studies have reported higher death rates of male patients with heart failure than death rates of their female counterparts [27,29-32]. That association was evident in the 1995 cohort, but greatly weakened in the 2000 cohort. Stated differently, the weaker secular trend in survival of female patients with CHF than male patients (Figure 1 and Table 2) may reciprocally be viewed as narrowing of the sex gap in survival over time (Table 3). The reasons for the latter trend remain speculative. Possible explanations include secular trends in co-morbidity, pharmacological therapy, and severity of CHF, which were not captured in the models. For example, it is possible that secular trends in CHF with preserved LVEF were different in men and women.

Three methodological difficulties have challenged attempts to describe the epidemiology of heart failure. First, heart failure is a syndrome – not a morphologically defined pathology, and therefore it is not always simple to diagnose this condition. Second, numerous definitions have been proposed for epidemiological research [33-35]. Third, many patients are diagnosed and treated in outpatient clinics, a setting that is less accessible to population-based research. Such limitations are inherent in every epidemiological research of secular trends in CHF burden, including the present work.

Some types of cases were not available to this study: patients who were not hospitalized for heart failure in the target year; symptom-free people with left ventricular systolic or diastolic dysfunction [36,37]; and hospitalized patients with heart failure but no sampled code [38]. It is likely, however, that these categories of missed cases were skewed toward mild forms of the syndrome and should not have greatly affected survival analysis of patients with moderate-to-severe CHF. Finally, after 1995 regulatory changes have put greater constraint on the use of medical records for research, so we could not access some selected records from 2000. Preliminary and sensitivity analyses, however, showed no major distortion of the 2000 sample.

## Conclusion

This analysis provides evidence for a favorable secular trend in survival of male patients following a CHF-related hospitalization. The reasons for modification of the secular trend by sex (or alternatively, modification of the sex effect by time-related factors) remain speculative. Documented benefits of ACE inhibitors and beta-blockers were evident in these observational data in both men and women.

## Competing interests

The author(s) declare that they have no competing interests.

## Authors' contributions

ES contributed to the design and conduct of the study, developed the analysis plan, and drafted the manuscript. SL established the database, contributed to the analysis plan, analyzed the data, and commented on the manuscript. Both authors read and approved the final manuscript.

## Pre-publication history

The pre-publication history for this paper can be accessed here:



## Figures and Tables

**Table 1:** Characteristics of hospitalized heart failure patients, by sex and year (1995 and 2000)

**Variable**	**Men**		**Women**	
	
	1995	2000	1995	2000
	(N = 1,216)	(N = 987)	(N = 1,041)	(N = 838)
Age (years, mean ± SD)	67 ± 10	66 ± 11	68 ± 9	68 ± 10
Ever Smoker (%)	68	72	53	57
LVEF (%, mean ± SD)	33 ± 15	33 ± 15	41 ± 17	41 ± 17
Missing (%)	54	46	56	50
Documented Medical History (%)				
Hypertension	62	68	67	74
Diabetes	35	37	36	40
Coronary Artery Disease	59	63	43	51
Congestive Heart Failure	65	69	59	70
In-Hospital Medication (%)				
ACE Inhibitor	62	65	57	55
Beta Blocker	19	54	20	50
Spironolactone	3	22	3	17
Discharge Medication (%)				
ACE Inhibitor	53	56	47	47
Beta Blocker	13	45	13	42
Spironolactone	2	19	1	16
Length of Hospital Stay (days, median, Q1–Q3)	5,3–8	5,3–8	5,3–9	5,3–8
Mortality (%)				
30 days	14	9	10	9
6 months	27	21	21	18
1 year	36	28	27	27

LVEF = left ventricular ejection fraction;

ACE = angiotensin converting enzyme

**Table 2:** Sex-specific hazard ratios of death within one year of a heart failure-related hospitalization

	**Hazard Ratio (95% Confidence Interval)**				
	
Variable	Model 1	Model 2	Model 3	Model 4	Model 5
	**Men (N = 2,203)**				
	
Year 2000 (vs. 1995)	0.76 (0.65–0.88)	0.77 (0.67–0.90)	0.75 (0.65–0.88)	0.75 (0.65–0.88)	0.82 (0.70–0.96)
Age (per 1 year)	--	1.03 (1.02–1.04)	1.03 (1.02–1.04)	1.03 (1.02–1.04)	1.03 (1.02–1.04)
History of CHF	--	--	1.61 (1.35–1.91)	1.67 (1.41–1.99)	1.56 (1.31–1.85)
ACE inhibitor (in hospital)	--	--	--	0.52 (0.45–0.60)	--
Beta blocker (in hospital)	--	--	--	--	0.76 (0.63–0.90)
	**Women (N = 1,879)**				
	
Year 2000 (vs. 1995)	0.99 (0.83–1.18)	1.00 (0.84–1.19)	0.96 (0.80–1.14)	0.93 (0.78–1.11)	1.13 (0.94–1.36)
Age (per 1 year)	--	1.03 (1.02–1.04)	1.03 (1.02–1.04)	1.03 (1.02–1.04)	1.03 (1.02–1.04)
History of CHF	--	--	1.44 (1.18–1.75)	1.45 (1.20–1.77)	1.40 (1.15–1.70)
ACE inhibitor (in hospital)	--	--	--	0.61 (0.51–0.73)	--
Beta blocker (in hospital)	--	--	--	--	0.56 (0.45–0.69)

ACE = angiotensin converting enzyme

**Table 3:** Year-specific hazard ratios of death within one year of a heart failure-related hospitalization

		**Hazard Ratio (95% Confidence Interval)**					
		**Men**		**Women**		**All**	
Variable		1995 (N = 1,216)	2000 (N = 987)	1995 (N = 1,041)	2000 (N = 838)	1995 (N = 2,257)	2000 (N = 1,825)
Sex	Women	--	--	--	--	Ref.	Ref.
	Men	--	--	--	--	1.41 (1.21–1.64)	1.09 (0.91–1.30)
Age (years)	35–64	Ref.	Ref.	Ref.	Ref.	Ref.	Ref.
	65–74	1.22 (0.96–1.55)	1.87 (1.37–2.55)	1.68 (1.20–2.33)	2.57 (1.76–3.75)	1.38 (1.14–1.68)	2.13 (1.67–2.70)
	75–79	1.89 (1.47–2.42)	2.32 (1.70–3.18)	1.92 (1.37–2.70)	2.44 (1.65–3.60)	1.90 (1.56–2.33)	2.35 (1.84–2.99)
LVEF (%)	>45	Ref.	Ref.	Ref.	Ref.	Ref.	Ref.
	26–45	0.67 (0.43–1.03)	1.29 (0.77–2.16)	1.98 (1.24–3.17)	1.30 (0.81–2.10)	1.11 (0.81–1.53)	1.25 (0.89–1.76)
	≤25	1.32 (0.88–1.96)	1.59 (0.97–2.60)	1.83 (1.08–3.10)	1.24 (0.76–2.02)	1.62 (1.18–2.22)	1.43 (1.03–2.00)
	Missing	1.20 (0.83–1.72)	1.78 (1.12–2.80)	1.88 (1.24–2.84)	1.67 (1.16–2.41)	1.52 (1.16–2.00)	1.70 (1.28–2.26)

LVEF = left ventricular ejection fraction
